# FDG-PET Response Prediction in Pediatric Hodgkin’s Lymphoma: Impact of Metabolically Defined Tumor Volumes and Individualized SUV Measurements on the Positive Predictive Value

**DOI:** 10.3390/cancers7010287

**Published:** 2015-01-28

**Authors:** Amr Elsayed M. Hussien, Christian Furth, Stefan Schönberger, Patrick Hundsdoerfer, Ingo G. Steffen, Holger Amthauer, Hans-Wilhelm Müller, Hubertus Hautzel

**Affiliations:** 1 Department of Nuclear Medicine (KME), Forschungszentrum Jülich, Medical Faculty, Heinrich-Heine-University Düsseldorf, Jülich, 52426, Germany; E-Mails: amrelsayed79@yahoo.com (A.E.M.H.); nuk@uni-duesseldorf.de (H.-W.M.); 2 Department of Nuclear Medicine, Medical Faculty, Heinrich-Heine-University Düsseldorf, Düsseldorf, 40225, Germany; 3 Department of Radiology and Nuclear Medicine, Medical School, Otto-von-Guericke University Magdeburg, Magdeburg, 39120, Germany; E-Mails: christian.furth@med.ovgu.de (C.F.); ingo.steffen@charite.de (I.G.S.); holger.amthauer@med.ovgu.de (H.A.); 4 Department of Pediatric Oncology, Hematology and Clinical Immunology, University Children’s Hospital, Medical Faculty, Heinrich-Heine-University Düsseldorf, Düsseldorf, 40225, Germany; E-Mail: stefan.schoenberger@ukb.uni-bonn.de; 5 Department of Pediatric Oncology and Hematology, Charité Campus Virchow, Humboldt-University Berlin, Berlin, 13353, Germany; E-Mail: patrick.hundsdoerfer@charite.de

**Keywords:** FDG-PET, pediatric Hodgkin lymphoma, response assessment, SUV

## Abstract

*Background*: In pediatric Hodgkin’s lymphoma (pHL) early response-to-therapy prediction is metabolically assessed by (18)F-FDG PET carrying an excellent negative predictive value (NPV) but an impaired positive predictive value (PPV). Aim of this study was to improve the PPV while keeping the optimal NPV. A comparison of different PET data analyses was performed applying individualized standardized uptake values (SUV), PET-derived metabolic tumor volume (MTV) and the product of both parameters, termed total lesion glycolysis (TLG); *Methods*: One-hundred-eight PET datasets (PET1, *n* = 54; PET2, *n* = 54) of 54 children were analysed by visual and semi-quantitative means. SUVmax, SUVmean, MTV and TLG were obtained the results of both PETs and the relative change from PET1 to PET2 (Δ in %) were compared for their capability of identifying responders and non-responders using receiver operating characteristics (ROC)-curves. In consideration of individual variations in noise and contrasts levels all parameters were additionally obtained after threshold correction to lean body mass and background; *Results*: All semi-quantitative SUV estimates obtained at PET2 were significantly superior to the visual PET2 analysis. However, ΔSUVmax revealed the best results (area under the curve, 0.92; *p* < 0.001; sensitivity 100%; specificity 85.4%; PPV 46.2%; NPV 100%; accuracy, 87.0%) but was not significantly superior to SUVmax-estimation at PET2 and ΔTLGmax. Likewise, the lean body mass and background individualization of the datasets did not impove the results of the ROC analyses; *Conclusions*: Sophisticated semi-quantitative PET measures in early response assessment of pHL patients do not perform significantly better than the previously proposed ΔSUVmax. All analytical strategies failed to improve the impaired PPV to a clinically acceptable level while preserving the excellent NPV.

## 1. Introduction

In oncology, volumetric assessment of response to therapy is based on CT as it forms a reproducible, easy-to-acquire rational for treatment evaluation. However, assessment of response-to-therapy in adult and pediatric Hodgkin lymphoma patients by positron-emission-tomography (PET) using the tracer F-18-fluorodeoxyglucose (FDG) has deemed an even more powerful tool for the discrimination of responders from non-responders [1,2,3] Thus, the incorporation of information provided by PET into the “Revised Response Criteria in Lymphoma” was inevitable [4,5]. In addition to the proposed visual PET reading the authors stated that semi-quantitative assessment, for example using the “standardized uptake value” (SUV), may provide a more uniform and potentially more accurate assessment of midtherapy PET (synonym to interim PET) studies. In this respect, we were able to demonstrate in a subset (*n* = 33/54) of the patients investigated in the present study, that the most accurate response-to-therapy assessment in pediatric Hodgkin lymphoma (pHL) was the percentaged decrease comparing the maximal SUV from pre-treatment PET to that of interim PET (*i.e.*, ΔSUVmax) [6]. With the requirement to warrant a negative predictive value (NPV) of 100% in response assessment, the data collected there indicated that early response assessment by PET using the ΔSUVmax achieved a positive predictive value (PPV) superior to visual PET assessment [6,7]. However, as the response to therapy assessment by both qualitative and semi-quantitative SUV-based approaches yielded only a moderate PPV at best [6,7,8,9] a more reliable metabolic and/or combined metabolic-morphologic index is imperative in order to optimize the correct prediction of responders *and* non-responders to therapy.

So far, the use of SUV neglects a lesion’s dimensions and the composition of the affected nodal and/or extra-nodal site, respectively. In other malignancies like mesotheliomas or oesophageal carcinomas a metabolically defined tumour volume (MTV) was identified as an independent prognostic factor for survival and as an even better predictor than SUVmax [10,11]. Additionally, in non-Hodgkin’s disease a combined CT/PET tumour burden calculation was proposed as a favourable index for treatment response assessment [12]. Hence, incorporation of MTV or calculation of the product of SUV and MTV, *i.e.*, the total lesion glycolysis (TLG), appears to be a promising tool for response prediction in pHL as well. Studies revealed that SUVs derived from larger fixed volumes-of-interest (VOI) are more reproducible than single-pixel-based SUVs [13,14,15,16,17]. SUV normalized to lean body mass and corrected to background (SUL) is preferred over SUV normalized to body weight (bw) or body surface area (SUV_bsa), as the former is more consistent from patient to patient [13,14,16,18,19,20]. In this context of individualized background correction the liver is reported to be especially well suited as it demonstrates little variance in FDG-accumulation [14,17,21].

Therefore, aim of this prospective multicentre study in pHL was to determine the value of PET-guided volume measurements (*i.e*., MTV, TLG) and individually background corrected parameters (SUL) in comparison to the visual assessment according to the Deauville-criteria [22]. Furthermore, regarding the positive predictive value the findings of the PET-guided volumes were compared to the ΔSUVmax, which right now is the best performing semi-quantitative PET-based parameter for response prediction in pHL.

## 2. Materials and Methods

### 2.1. Patients

Between November 2002 and January 2010, 54 patients (male, *n* = 33; female, *n* = 21; mean age, 14.6 (range, 5.9 to 17.8) years) with histopathologically proven HL from four institutions were enrolled in this prospective study (Table 1). Thirty-three of these patients were included in the PET2003 multicentre study [6,7,8]. The study was carried out in accordance with the Declaration of Helsinki and the principles of good clinical practice. Approval was granted by the local institutional review board and the German Federal Office for Radiation Protection (Bundesamt für Strahlenschutz). Exclusion criteria were patients with other malignancy (double primary), life-threatening impairment of organ function, pregnancy, diabetes mellitus, or age younger than 1 year and older than 18 years at date of initial diagnosis. Written informed consent was obtained from all patients and/or parents. Staging was performed according to the Ann Arbor classification [23] and all patients were subsequently stratified to the appropriate therapy groups (TG): TG1, stage IA/B to IIA; TG2, stage IEA to IIIA; TG3, stage IIEB to IVB) and treated according to the appropriate treatment optimization protocol (TOP) (GPOH-HD2002P, GPOH-HD2003, EuroNet-PHL-C1) [24,25,26,27,28]. In addition to conventional imaging methods (CIM) all patients underwent two FDG-PET or FDG-PET/CT examinations: PET1 before administration of any anti-neoplastic treatment and PET2 after completion of the first two cycles of chemotherapy. PET2 was carried out as close as possible to the third cycle of chemotherapy (range, 14–17 d after end of 2nd cycle). Involved-field radiotherapy was performed after completion of chemotherapy in 41 of 54 patients according to the appropriate TOP. Three patients received further radiotherapy after diagnosis of recurrence. Mean follow-up was 65 months (range, 27 to 112 months).

**Table 1:** Patients characteristics.

Characteristics	No. of Patients (*n* = 54)
Sex Male	33
Female	21
Age mean (years)	14.6
range (years)	5.9–17.8
Histology	
Nodular sclerosis (n)	38
Mixed cellularity (n)	13
Lymphocyte rich (n)	2
no final subtype (n)	1
Stage	
I (n)	3
II (n)	26
III (n)	12
IV (n)	13
Extra-nodal disease	20
Therapy groups	
TG1 (n)	17
TG2 (n)	13
TG3 (n)	24
Follow-up (months)	65
Range (months)	27–112
Recurrences	6

Abbreviation: TG, therapy group.

### 2.2. PET: Acquisition

Whole-body PET scans were acquired according to the recommendations of the “European Association of Nuclear Medicine” guidelines (protocol details are published elsewhere [29]). All patients were euglycemic (range, 60 to 120 mg/dL). Starting 71.9 ± 20.5 min (PET1) and 68.7 ± 23.5 min (PET2), respectively, after FDG injection emission and transmission data were collected. Regarding the differences in scanner performance through this multicentre trial, appropriate scanner calibrations had been performed to minimize discrepancies. Scanner types and acquisition protocols are summarized in [7,8]. With respect to the included FDG-PET/CT studies, the acquired PET data were read alone and assessed independently from the CT scans.

### 2.3. Image Reconstruction

Maximum intensity whole body projections as well as coronal, axial and sagittal slices were reconstructed using dedicated PET software (rover^®^ v2.0.31, ABX GmbH, Radeberg, Germany). For semi-quantitative analyses attenuation-corrected data were used only.

### 2.4. Image Analysis

PET analyses were performed qualitatively (I) and by means of semi-quantitative measures (II).

#### 2.4.1. Visual Analysis

The visual response-to-therapy assessment was done in consensus by two experienced nuclear medicine specialists (Amr Elsayed M. Hussien, Hubertus Hautzel) blinded to the corresponding CIM and clinical results and in accordance to the Deauville-criteria [22]. In each patient, 23 lymph node regions and 9 extra-nodal regions were evaluated for the presence of lymphoma tissue using a standardized report form. PET-negative ratings at interim were expressed as score 1 or 2, respectively, whereas PET-positive ratings at interim were characterized by a score 3, 4 or 5, respectively.

#### 2.4.2. Semi-Quantitative Analysis

In PET1, the areas containing visually detected lymphoma lesions were localized using masks with different shapes (spherical, rectangular or cylindrical, respectively, whichever were more appropriate to be best suited to cover the lesions’ shapes). To create irregular isocontour VOIs delineating the lesions with pathologically increased FDG-uptake within these predefined masks, three different thresholds were used: (1) Fixed threshold SUV 2.5 corrected to body weight (SUV). This threshold was chosen in accordance with the suggestion for the use of the PERCIST criteria in the therapy response assessment of solid tumours [6,19]; (2) fixed threshold SUV 2.5 corrected to bsa (SUV_bsa); (3) SUV corrected for lean body mass (lbm) and adjusted to background (SUL). The latter was calculated using a 5.15 cm diameter spherical VOI placed on the normal superior right lobe of the liver [19]. None of the patients demonstrated a hepatic involvement of the lymphoma therefore in all patients this individual thresholding was feasible. Mean liver SUL and its SD were measured. The threshold SUL was set to the mean liver SUL plus two standard deviations (SD) [19]. This threshold aimed to exclude the individual background contribution in the involved lesions.

Target lesions were all detectable lesions exceeding the predefined threshold. VOI-masking tools were used to precisely exclude areas of high but physiological FDG-uptake (e.g., brain, cardiac muscle, urinary tract) from relevant pathological uptake. In the semi-quantitative analyses suspected brown fat was separated from pathological uptake by an anatomical correlation of the FDG uptake with the appropriate CIMs. For each type of the above mentioned thresholds different irregular isocontour VOIs were created in PET1. Various parameters were quantitatively assessed by help of these VOIs: (1) maximal SUV (SUVmax1, SUVmax1_bsa); (2) mean SUV (SUVmean1, SUVmean1_bsa) and (3) metabolic tumour volume (MTV1_bw, MTV1_bsa). To estimate the latter the above mentioned fixed threshold of 2.5 (MTV1_bw, MTV1_bsa) were applied for volume rendering. Those masks derived from PET1 were identically placed in the PET2 datasets to measure the same parameters as in PET1: (1) maximal SUV (SUVmax2, SUVmax2_bsa); (2) mean SUV (SUVmean2, SUVmean2_bsa) and (3) metabolic tumour volume (MTV2_bw, MTV2_bsa). Multiplicative combination of metabolic (*i.e.*, mean uptake values) and volumetric (*i.e.*, MTV) measures of the SUV, the SUV_bsa and the SUL analyses were also calculated in order to test if the combined metabolic/anatomical information, that means the TLG, may yield superior results: (1) TLGmean (SUVmean × MTV_bw); (2) TLGmean_bsa (SUVmean_bsa × MTV_bsa); (3) TLGmean_SUL (SULmean × MTV_SUL). Measures on TLG were obtained at PET1 and PET2 (e.g., TLGmean1, TLGmean2, *etc.*).

The relative changes of all values between PET1 and PET2 were computed as the decrease in percentage [e.g., ΔSUVmax = (SUVmax1-SUVmax2)/SUVmax1 × 100)].

### 2.5. Standard of Reference

To create a standard of reference, the results of CIMs and FDG-PET were finally verified by an interdisciplinary tumour board. For verification of lesion status, all staging and follow-up examinations, histopathology of biopsies, and clinical data including the serial follow-ups were used. All patients underwent regular follow-up investigations including physical examinations, blood tests, chest x-ray and ultrasound quarterly during the first year, half yearly during the second year, and once a year thereafter. Suspicion of relapse was confirmed by biopsy.

### 2.6. Sample Size Estimation

The main goal of testing alternative strategies of PET analysis was to increase the PPV of FDG-PET from the 60% recently reported [6,9] to at least 80% in order to draw reliable therapeutic decisions from a positive result. At the same time the already optimal NPV of 100% should be maintained. To reliably answer this question by statistical means a sample size estimation was performed (Statistica v6.0, StatSoft Inc., Tusla, OK, USA). The main goal was predefined as an increase in PPV from 60% to 80%. Using a one-tailed chi-square test, fixing the type I error (alpha) to 0.05 and setting the statistical power to at least 90% the sample size estimation resulted in a group size n of at least 44 patients yielding an actual power of 91.42%.

### 2.7. Statistical Analysis

Regarding the qualitative visual rating of the interim PET, patients with CR were classified as true negative (TN) if PET2 was negative (score 1 and 2) and as false positive (FP) if PET2 was considered positive (score 3, 4 or 5, respectively) but the respective patient was in ongoing remission at the time point of analysis. Patients with recurrence during follow-up and classified to be PET-positive at PET2 (score ≥ 3) were referred to be true positive (TP). Patients were referred to be false negative (FN) if PET2 was considered negative (score 1 and 2) and recurrence of disease was detected during follow-up.

Subsequent data analysis was done using the Analysis-it software^®^ (http://www.analyse-it.com). Sensitivity, specificity, accuracy, PPV and NPV were calculated after using receiver operator characteristics (ROC)-curves to assess the cut-off points for the most accurate response-to-therapy prediction. For the ROC-derived areas under the curve (AUC) P-values were calculated. *p*-values < 0.05 were regarded as significant. As multiple testing was carried out, the p-value was adjusted accordingly by use of the false discovery rate (FDR) correction. This FDR-correction resulted in a final *p*-value of < 0.006 in order to control for an overall α error of 5% or less. To identify the best performing strategy of the semi-quantitative analyses the AUCs were statistically compared by use of the DeLong-DeLong-Clarke-Pearson covariance matrix [30].

In addition, to test the response prediction of lesion uptake (SUVmax2/SUVmean2) and lesion size (MTV2) a logisitic regression was calculated (Statistica v6.0).

## 3. Results

Six of 54 patients had a recurrence of the disease (time to relapse: 3 months, *n* = 2; 8 months, *n* = 1; 16 months, *n* = 2; 20 months, *n* = 1). The remaining 48 patients were in CR at time of analysis.

### 3.1. Visual Assessment

Qualitative response assessment by visual ratings of PET2 revealed a sensitivity of 66.7% and a specificity of 70.8% (AUC, 0.69; *p*-value, 0.044 (not significant after FDR-correction for multiple testing); PPV, 22.2%; NPV, 94.4%; accuracy, 70.4%) (Table 2).

### 3.2. Semi-Quantitative Assessment—Response Prediction Using PET1

The AUC-analyses of all PET1-based parameters yielded no significant results; therefore the PET1 results were not further considered [31].

### 3.3. Semi-Quantitative Assessment—Response Prediction Using PET2

**Figure 1:**
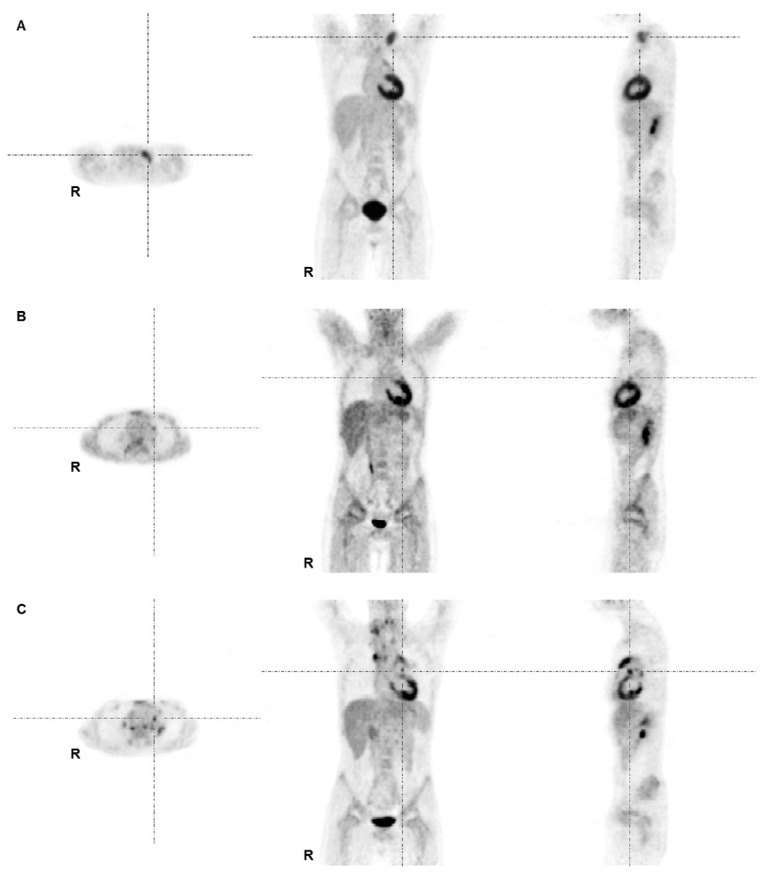
FDG-PET images of an 11-year-old male patient with mixed cellularity HL stage II at staging (**A**), interim (**B**) and at restaging due to suspected relapse 16 months after initial diagnosis (**C**). (**A**) Lymphoma lesions at staging were found at left supra- and infraclavicular sites and in the mediastinum. The patient was treated according to the TG1 protocol (GPOH-HD2002P); (**B**) The interim PET demonstrated minimal residual uptake in the left mediastinum adjacent to the left atrium/ventricle primarily interpreted as physiological myocardial uptake (with no anatomical equivalent in the corresponding low-dose CT). This lesion was missed by the first truth-panel assessment (false negative by visual assessment). By semi-quantitative means the lesion exceeded SUV thresholds (true positive by semi-quantitative means); (**C**) PET at time point of restaging showed multiple areas of intense tracer uptake indicating a recurrence.

**Figure 2:**
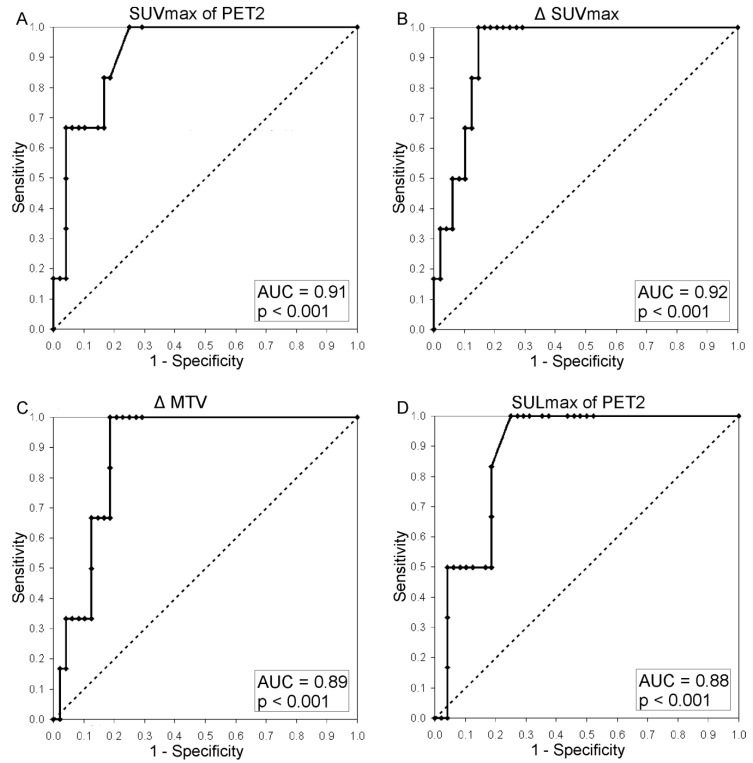
Receiver operating characteristics-curve of (**A**) SUVmax of PET2; (**B**) ΔSUVmax, (**C**) ΔMTV using a SUV of 2.5 as cut-off for PET1 and PET2 data; (**D**) SULmax of PET2.

For clarity a comprehensive overview is given in Table 2. Additional statistics for all parameters derived from PET2 are given in supplementary Table 1. Both SUVmax2 and SUVmean2 performed significantly better than the qualitative response assessment (SUVmax2 *vs.* visual: *p* = 0.034; SUVmean2 *vs.* visual: *p* = 0.04). The same was true for the SUV-derived MTV and TLG results of PET2. Figure 1 illustrates a patient considered to be TP at PET2 when assessed by semi-quantitative means who was FN in the visual assessment. The best performing AUC at PET2 was found for SUVmax2 (AUC, 0.91) (Table 2 and Figure 2). While keeping the optimal NPV of 100% SUVmax2, SUVmean2 and TLGmean2 yielded a PPV of 33.3%. The performance of the SUL parameters individually corrected for lean body mass and background was not superior to the results of the SUV-based data (see Table 2 and Figure 2). All body-surface-area-corrected SUV-parameters from PET2 were not capable in separating responders from non-responders. Thus, the body-surface-area-corrected were not further evaluated.

### 3.4. Semi-Quantitative Assessment—Response Prediction Using Δ-Analyses (PET1-PET2)

A comprehensive overview is given in Table 2. Regardless of the parameters used for the PET1 *vs.* PET2 analyses (Δ) all SUV estimates as well as the derivatives thereof were suitable to separate responders from non-responders. In comparison to the visual assessment all ΔSUV-based analyses performed significantly better. Highest AUC was seen for ΔSUVmax (AUC, 0.92; *p* < 0.001; sensitivity, 100%; specificity, 85.4%; PPV, 46.2%; NPV, 100%; accuracy, 87.0%) (Figure 2), followed by ΔSUVmean, ΔTLGmax and ΔTLGmean (all with AUC of 0.90). Again, the body-surface-area-corrected Δ-analyses were not capable of identifying repsonders and non-responders therefore omitted in subsequent statistics.

**Table 2:** Single time point analyses and Inter-time point analyses comparing relative differences between PET1 and PET2: Results for response prediction at interim PET (PET2) and Δ-analyses using receiver operating characteristics.

Visual Assessment		No.	AUC	95% CI (%)	*p* Value	Cut-off (Absolute)	Sensitivity (%)	Specificity (%)	PPV (%)	NPV (%)	TP	TN	FP	FN	Accuracy (%)
		54	0.69	0.47	0.044 (n.s.)		66.7	70.8	22.2	94.4	4	34	14	2	70.4
SUV	SUVmax2	54	0.91	0.83–1	<0.0001	>2.5	100	75.0	33.3	100	6	36	12	0	77.8
	SUVmean2	54	0.90	0.80–0.99	<0.0001	>2.5	100	75.0	33.3	100	6	36	12	0	77.8
	MTV2	54	0.90	0.80–1	<0.0001	>0 mL	100	70.8	30.0	100	6	34	14	0	74.1
	TLGmean2	54	0.90	0.81–1	<0.0001	>0.3 g	100	75.0	33.3	100	6	36	12	0	77.8
ΔSUV	ΔSUVmax	54	0.92	0.85–1	<0.0001	<78.0%	100	85.4	46.2	100	6	41	7	0	87.0
	ΔSUVmean	54	0.90	0.81–0.98	<0.0001	<48.0%	100	81.3	40.0	100	6	39	9	0	83.3
	ΔMTV	54	0.89	0.79–0.98	<0.0001	<99.9%	100	81.3	40.0	100	6	39	9	0	83.3
	ΔTLGmean	54	0.90	0.81–0.99	<0.0001	<99.95%	100	81.3	40.0	100	6	39	9	0	83.3
SUL	SULmax2	54	0.88	0.78–0.98	<0.0001	>2.1	100	75.0	33.3	100	6	36	12	0	77.8
	SULmean2	54	0.86	0.76–0.97	<0.0001	>1.8	83.3	85.4	41.7	97.6	5	41	7	1	85.2
	MTV2_liver_cor.	54	0.81	0.65–0.98	<0.0001	>4.1 mL	83.3	77.1	31.3	97.4	5	37	11	1	77.8
	TLGmean2_liver_cor.	54	0.83	0.67–0.99	<0.0001	>0 g	100	47.9	19.4	100	6	23	25	0	53.7
ΔSUL	ΔSULmax	54	0.86	0.75–0.98	<0.0001	<83.3%	100	66.7	27.3	100	6	32	16	0	70.4
	ΔSULmean	54	0.84	0.73–0.95	<0.0001	<45.2%	100	75.0	33.3	100	6	36	12	1	77.8
	ΔMTV_liver_cor.	54	0.79	0.63–0.95	0,0002	<99.9%	100	56.3	22.2	100	6	27	21	0	61.1
	ΔTLGmean_liver_cor.	54	0.79	0.62–0.95	<0.0003	<99.93	100	56.3	22.2	100	6	27	21	0	61.1

Abbreviations: Δ, delta signal reduction of PET2 compared to PET1 expressed in percent; No., number of patients included for analyses; AUC, area under the curve; CI, confidence interval; PPV, positive predictive value; NPV, negative predictive value; TP, true positive; TN, true negative; FP, false positive; FN, false negative; n.s., not significant after FDR correction for multiple comparisons, SUV, standardized uptake value; max, maximal; SUL, standardized uptake value, lean body mass and background (liver) corrected; MTV, metabolic tumor volume; TLG, tumor lesion glycolysis.

The DeLong-DeLong-Clarke-Pearson covariance matrix revealed no statistically significant superiority of one of the SUV-derived semi-quantitative analytical strategies over the others.

**Figure 3:**
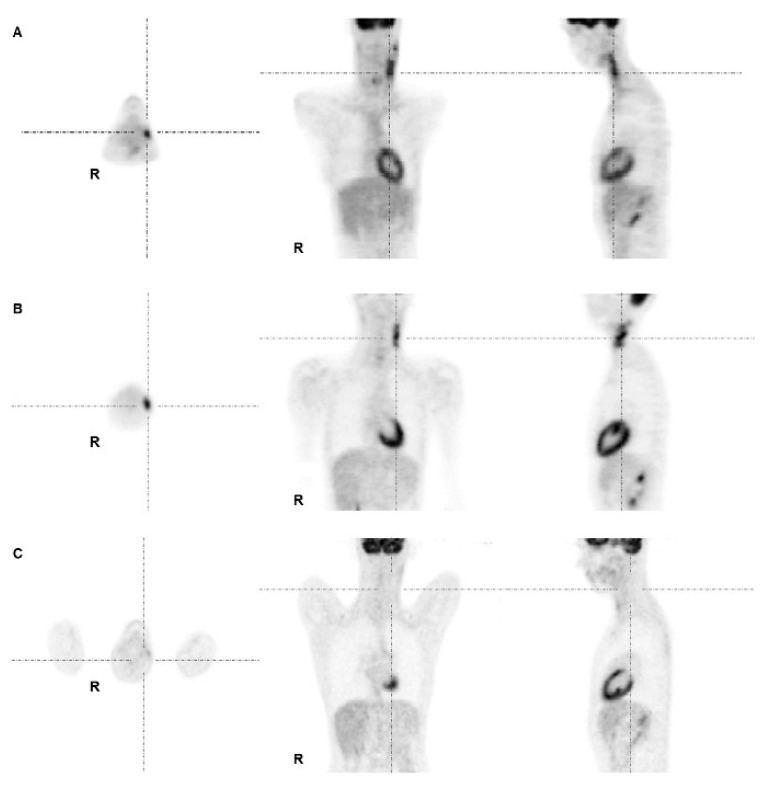
PET images of a 17-year-old male patient with lymphocyte rich HL stage I. (**A**) Lymphoma was initially localized in left cervical LN, and was treated according to TG1; (**B**) PET2 after two cycles of chemotherapy demonstrated a large residual uptake at the left cervical region with SUVmax reaching 8.2; (**C**) PET3 after end of chemotherapy showed disappearance of any tracer uptake at the corresponding site. The subsequent follow-up was uneventful, the patient is still in complete remission. Therefore, PET2 was rated false positive.

Finally, using an optimal cut-off of 78% decrease in FDG uptake from PET1 to PET2 in the ΔSUVmax-analysis all patients suffering relapse were separated from responding patients. However, this cut-off yielded seven FP cases (Figure 3) while it maintained the rate of no FN results. Thus, the achievable PPV in Δ-analyses was 46.2% at maximum.

In order to calculate a predictive model, the logistic regression demonstrated a significant impact on the final treatment outcome for SUVmax2 (*p* = 0.016) while MTV2 and SUVmean2 or a parameter combination were not significant.

## 4. Discussion

To achieve the goals of TOPs in patients suffering from pHL an optimal NPV of the applied diagnostics up to 100% is of paramount importance to minimize FN results—ideally to zero—and thus avoiding unexpected recurrences. Using a sensitive, merely visual read out FDG-PET almost provides such a high NPV with 94.4%. This NPV reaches 100% by the additional use of semi-quantitative analyses. In contrast, the PPV of FDG-PET in pHL is impaired reaching 60% at its best [6,9]. Like previous studies [6,7], which incorporated 33/54 of the patients studied here, our results are in favour of semi-quantitative tools over visual assessment but resulting PPV still remains poor. However, the qualitative reading of the images is still a valuable cornerstone of staging for defining those anatomical regions where to place VOIs for semi-quantitative lesion assessment and for easily judging PET2 scans as negative.

With respect to the semi-quantitative analyses, PET1 results were not capable of separating responders from non-responders in general. However, PET2 analyses and Δ-PET1/PET2 analyses were significant in defining responders and non-responders to therapy with an accuracy of up to 87.0%. Comparing the ROCs of all semi-quantitative PET2 measures gathered in Table 2 no significant differences between them were apparent with respect to their AUCs and p-values. The additional correction of the SUV data for lean body mass and individual background had no further impact on the discrimination of responders from non-responders. Thus, all proposed measures could be used interchangeably according to the preference of the user but less time consuming semi-quantitative analyses might be favoured. The logistic regression demonstrated that only SUVmax2, but not SUVmean2 or MTV2 or a combination of these parameters is predictive for the latter outcome. In general, the overall performance of the PET2 SUVmax result is the fastest and most easily achievable semi-quantitative measure to identify all subsequent responders, but at the price of a low PPV (33.3%). Considering our main goal—to improve the PPV while keeping an optimal NPV of 100%—none of the analytical approaches reached the initially intended goal of a PPV of at least 80%. Also the addition of PET1 and calculation of the ΔSUVmax-estimates led only to a slight PPV improvement to 46.2%, still not sufficient to rely on for clinical decisions. Even when restricting analyses to patients with higher initial stages or extra-nodal disease therefore receiving an intensified treatment (*i.e.*, TG2, TG3), PPV of the ΔSUVmax analysis increased only to 50% (TP = 5, FP = 5). The addition of the metabolically defined volume change resulting in ΔMTV- or ΔTLG-estimates did not add to a further increase in PPV. These findings contradict our initial proposition that entire viable tumour burden measured by volumetric evaluation techniques might yield better results than only assessing the most metabolically active part of the tumour. In addition, our tumour volume-based results are in contrast to those previously demonstrated in non-Hodgkin’s lymphoma [12]. Therefore, from our data perspective these elaborated and time-consuming volumetric strategies cannot be recommended for the response assessment in pHL patients.

In general, our results are in support of the widely-used SUVmax-based parameters as a de-facto standard in semi-quantitative FDG-PET analysis and provide evidence that the most active and aggressive part of the tumour portrays the relevant metabolic state of heterogeneous lymphoma tissue [6], irrespective of the error sources of such a single voxel measurement (e.g., patient preparation, uptake time, scanner errors, image reconstruction and filtering, VOI definition) [13,32,33,34]. This semi-quantitative SUVmax estimation extends the qualitative approach in a direct line as the visual assessment of PET scans in pHL patients also rates a focal FDG uptake by comparison to the mediastinal blood pool structures and to the liver [6,7], thus not accounting for additional heterogeneous low intensity uptake in lymphoma tissue. Hasenclever and colleagues proposed a so-called SUVpeak averaging the values of that voxel carrying the maximum SUV and those of the three hottest adjacent voxels and compared this SUVpeak to the average liver SUV in order to achieve an intra-subject background correction [35]. The authors argued that this semi-quantitative approach is less rater-dependent than using the Deauville criteria and, due to the continuum of outcomes instead of the stepwise Deauville scores, might yield superior results with respect to identifying true positive cases while minimizing false positive ones, *i.e.*, increasing the PPV. Since their follow-up data was too sparse at date of data analysis they were not able to test this hypothesis. To question this, we extended our analyses by background-correcting SUVmax and SUVmean, respectively, by the liver’s mean uptake in the same way than Hasenclever *et al.* and as proposed by Wahl *et al.* [19,35]. This approach resulted in the set of SUL data. However, even using these individually background corrected data all PET2 and ΔPET1/PET2 results failed to increase the PPV over that of ΔSUVmax.

With respect to the proposed maximal SUV values or relative decreases of SUVmax (*i.e.*, ΔSUVmax) our analyses might be limited due to the partial volume effect (PVE) as the true FDG uptake in the single hottest voxel is underestimated due to the PVE. This issue was addressed in the context of oncological FDG PET studies for staging and patient monitoring (see [36] for review). While correction for PVE significantly increases the resulting SUV data from patient studies seeking to demonstrate an additional value of PVE correction are ambiguous. While some studies ([37] in breast cancer, [38] in dignity differentiation of lung nodules) argued in favor of PVE correction others could not show a beneficial effect ([39] in lung nodules, [40] in non-small cell lung cancer; [41] in oesophageal cancer). Focusing on our main objective, early response-to-chemotherapy prediction, Maisonobe *et al.* investigated PVE correction in a comparable setting; specifically in patients with metastatic colorectal cancer after one course of chemotherapy [42]. In their study PVE correction did not improve the response prediction as compared to uncorrected PET data. Taken together, presently the gathered data are not unequivocally in support of the hypothesis that PVE correction might result in a substantial improvement of the predicitive values of SUVmax based PET analyses.

The low PPV of FDG PET in pHL in our and previous studies may be due to excellent cure rates of the current TOPs [25,27] leading to a limited number of recurrences. In terms of statistics this relates to a small number of true positive cases. On the other hand, by increasing the overall sample size the number of true negatives rises and also those of the false positives and true positives but at a much smaller rate. This results in an improved specificity; however, PPV remains poor as the increase in false positives is close to that of the true positives. Another aspect which might explain the overall low PPV of the interim PET and the Δ-PET1/PET2 analyses is that PET2 is obtained after a part of therapy (first two cycles of chemotherapy) while its results are compared to the final outcome of the whole treatment (successive chemotherapy courses and/or radiotherapy). The overall effects of this entire treatment might convert patients with still viable lymphoma tissue after two cycles of chemotherapy into complete remission at the end of therapy. Thus, those patients would be PET-positive at the interim PET which means true positive at that time but responders over the full treatment course. In this case, the PET2 result is retrospectively judged as false positive. To finally confirm this hypothesis biopsies from interim PET positive lesions would have to be taken. However, from an ethical point of view drawing multiple histologic specimens is questionable in children.

One might conclude that the high rate of false positive cases is not necessarily a weakness of FDG-PET but in turn is owed to the strength of the subsequent therapy and the disproportional time points of treatment success estimation. Considering this, a positive interim PET2 is not an optimal base to draw a treatment escalation decision from, *i.e.*, adding radiotherapy but indicates the need for additional molecular imaging. In this respect proliferation markers like ^18^F fluorothymidine (FLT) or L-[methyl-^11^C] methionine appear to complement this shortcoming of the FDG-PET diagnostics. Such a tracer combination in selected patients might contribute to a substantial improvement of the overall PPV as derived from molecular imaging in pHL.

One main goal of the consecutive multicentre TOPs in pHL was a reduction of late therapy side effects by elimination of radiotherapy for TG1 patients. However, despite a negative PET2 radiotherapy was omitted in TG1 patients of the GPOH-HD2003 study only if concomitant CIM revealed a 95% anatomical lesion reduction and a residual volume of less than 2 mL. In that TOP FDG-PET scans were compared to CIM as the gold standard and exclusion of radiotherapy was confined to TG1 only even if TG2 or TG3 patients had a complete metabolic interim PET response. Given the optimal NPV of PET2 and the aimed reduction of radiotherapy-induced late therapy effects, a strategy of therapy de-escalation in TG2 and TG3 was put forward in the EuroNet-PHL-C1 protocol [24,26]. Radiotherapy was avoided in four of our patients treated according to the EuroNet-PHL-C1 protocol despite their advanced initial stage (TG2, *n* = 2; TG3, *n* = 2). The reason for this de-escalation was an excellent response to the first two cycles of chemotherapy with a negative PET2 and an almost complete remission in CIM. These four patients remained in complete remission over the entire subsequent surveillance (follow up: mean, 37.5 months; range, 27–49 months). Other studies showed that omission of radiotherapy in advanced stage patients with excellent therapy response as assessed by CIM alone led to increased relapse rates [28,43]. This underlines the inferiority of CIM as compared to a negative interim PET in terms of predicting a favourable therapy outcome and emphasizes the use of PET2 to exclude radiotherapy in metabolically complete responders hence decreasing the incidence of adverse late therapy effects.

## 5. Conclusions

In summary, elaborated techniques of semi-quantitatively reading FDG-PET data for early response-to-therapy assessment in pHL such as SUV correction for body surface area or metabolically guided volumetric analyses (e.g., MTV, TLG) fail to improve the PPV to a clinically acceptable minimum of at least 80% while preserving maximum NPV. Instead, SUVmax estimation of the interim PET or—in order to increase the PPV—the ΔSUVmax from the staging PET to the interim PET demonstrate equivalent results whereas their calculation is much faster in a clinical setting. From a clinical point of view the high NPV provides an option to avoid radiotherapy in interim PET-negative pHL patients regardless of their initially attributed disease-related risk.
